# In Vitro Comparative Study of the Inhibitory Effects of Mangiferin and Its Aglycone Norathyriol towards UDP-Glucuronosyl Transferase (UGT) Isoforms

**DOI:** 10.3390/molecules22061008

**Published:** 2017-06-16

**Authors:** Dan Sun, Chun-Ze Zhang, Rui-Xue Ran, Yun-Feng Cao, Zuo Du, Zhi-Wei Fu, Chun-Ting Huang, Zhen-Ying Zhao, Wei-Hua Zhang, Zhong-Ze Fang

**Affiliations:** 1 College of Life Sciences, Nankai University, Tianjin 300071, China; sundan@nankai.edu.cn; 2 Department of Colorectal Surgery, Tianjin Union Medical Center, Tianjin 300121, China; zhangchunzetj@163.com; 3 Tianjin Key Laboratory on Technologies Enabling Development of Clinical Therapeutics and Diagnosis, School of Pharmacy, Tianjin Medical University, Tianjin 300070, China; ranruixue@tmu.edu.cn; 4 Key Laborotary of Liaoning Tumor Clinical Metabolomics (KLLTCM), Jinzhou 121001, Liaoning, China; caoyunfeng@dicp.ac.cn (Y.-F.C.); zsjz2113@sina.com (Z.D.); fffffzhiwei@163.com (Z.-W.F.); sunshiyang@126.com (C.-T.H.); 5 Department of Toxicology, School of Public Health, Tianjin Medical University, 22 Qixiangtai Road, Heping District, Tianjin 300070, China; 6 Tianjin Union Medical Center, 190 Jieyuan Road, Hongqiao District, Tianjin 300121, China; clinpharmzhao@163.com

**Keywords:** mangiferin, norathyriol, UDP-glucuronosyltransferases (UGTs), herb–drug interactions

## Abstract

Mangiferin (MGF), the predominant constituent of extracts of the mango plant *Mangifera Indica* L., has been investigated extensively because of its remarkable pharmacological effects. In vitro recombinant UGTs-catalyzed glucuronidation of 4-methylumbelliferone (4-MU) was used to investigate the inhibition of mangiferin and aglycone norathyriol towards various isoforms of UGTs in our study, which evaluated the inhibitory capacity of MGF and its aglycone norathyriol (NTR) towards UDP-glucuronosyltransferase (UGT) isoforms. Initial screening experiment showed that deglycosylation of MGF into NTR strongly increased the inhibitory effects towards almost all the tested UGT isoforms at a concentration of 100 μM. Kinetic experiments were performed to further characterize the inhibition of UGT1A3, UGT1A7 and UGT1A9 by NTR. NTR competitively inhibited UGT1A3, UGT1A7 and UGT1A9, with an IC_50_ value of 8.2, 4.4, and 12.3 μM, and a *Ki* value of 1.6, 2.0, and 2.8 μM, respectively. In silico docking showed that only NTR could dock into the activity cavity of UGT1A3, UGT1A7 and UGT1A9. The binding free energy of NTR to UGT1A3, 1A7, 1A9 were −7.4, −7.9 and −4.0 kcal/mol, respectively. Based on the inhibition evaluation standard ([I]/*Ki* < 0.1, low possibility; 0.1 < [I]/*Ki* < 1, medium possibility; [I]/*Ki* > 1, high possibility), an in vivo herb–drug interaction between MGF/NTR and drugs mainly undergoing UGT1A3-, UGT1A7- or UGT1A9-catalyzed metabolism might occur when the plasma concentration of NTR is above 1.6, 2.0 and 2.8 μM, respectively.

## 1. Introduction

**Figure 1:**
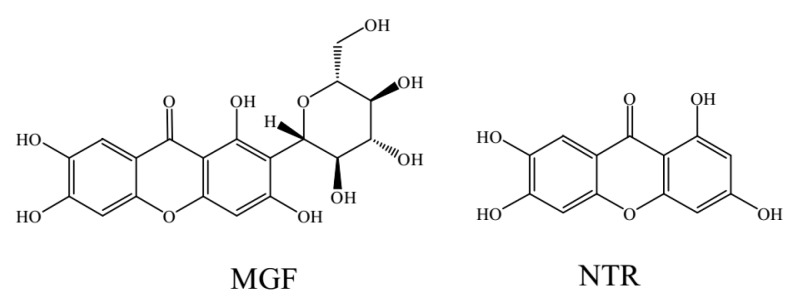
Chemical structure of mangiferin (MGF) and norathyriol (NTR).

Mangiferin (1,3,6,7-tetrahydroxyxanthone-C-2-β-d-glucoside, MGF, Figure 1), the predominant constituent of extracts of the mango plant *Mangifera Indica* L., has been investigated extensively because of its remarkable pharmacological effects, including antioxidant, anti-inflammatory, antidiabetic, anticancer, antibacterial, anti-HIV, radioprotective activities and promising diabetes treatment [1,2,3,4,5,6,7,8,9,10,11].

Pharmacokinetic results have revealed that mangiferin is absorbed rapidly and exhibits poor bioavailability (only 1.2%) in rat [12,13]. This poor bioavailability may be partially attributed to the low absorption, the hepatic first pass effect that is a phenomenon of drug metabolism whereby the concentration of a drug is greatly reduced before it reaches the systemic circulation [14,15] and extensive metabolism of mangiferin. On the one hand, mangiferin itself underwent phase II metabolism to be converted into monomethylated, dimethylated, monoglucuronidated, diglucuronidated, monoglucuronidated, monoglucuronidated, monoglucuronidated and monosulfated conjugates. On the other hand, mangiferin was initially deglycosylated to be transformed into norathyriol, which was further subjected to methylation, reglycosylation, dehydroxylation, glucuronidation, and sulfation [16]. These metabolites were then absorbed into serum and transferred into various organs to exert therapeutic effects [17]. The C-glucosyl bond of C-glucosides generally tolerates both acid and enzymatic hydrolysis. Thus, mangiferin was transformed into its aglycone norathyriol (1,3,6,7-tetrahydroxyxanthone, NTR, Figure 1) mainly by human intestinal bacteria [18,19]. Norathyriol is known to possess antioxidant, anti-inflammatory, and antitumor properties [20,21,22,23]. Furthermore, MGF and NTR could improve the glucose utilization and insulin sensitivity by up-regulation of the phosphorylation of AMPK [24].

Although many studies have been carried on the in vitro and in vivo metabolism profiles of MGF and NTR, only a few articles that deal with metabolism enzymes have elucidated the potential toxicity and herb-drug interactions. Goud et al. found that mangiferin showed modulation of certain P450 enzymes, the most important phase I metabolizing enzyme [25]. Nevertheless, compared with phase I metabolizing enzymes, no research had been carried out on the interaction of MGF and NTR with phase II conjugating enzymes.

UGTs (EC 2.4.1.17) are a family of conjugating enzymes that play important roles in the metabolism of endogenous and exogenous compounds [26]. UGT-catalyzed glucuronidation reactions are responsible for about 35% of all drugs metabolized by phase II enzymes [27]. A glucuronidation reaction catalyzed by UGT enzymes serves as an integral step in transforming lipophilic substrates into hydrophilic glucuronides, a process that increases their ability to partition into the aqueous intra- and extra-cellular compartments of the body, facilitating the transport to excretory organs and subsequent elimination through the bile and urine [27,28]. Thus, glucuronidation serves as an essential clearance and detoxification mechanism for endogenous and exogenous compounds [29,30].

Herb-drug interactions caused by UGT enzymes have received increasing attention over the past few decades. For example, sorafenib-induced UGT1A1 inhibition might also be an important reason for the elevation of serum bilirubin [31]. Fluconazole itself is not metabolized by UGT but alter pharmacokinetic parameters of co-administrated zidovudine in AIDS patients, because the glucuronidation of zidovudine by UGT2B7 is strongly inhibited by fluconazole [32]. Cycloastragenol has been proven to specifically inhibit UGT1A8 and UGT2B7, thus affecting drugs undergoing UGT1A8- and UGT2B7-catalyzed metabolism [33].

In vitro recombinant UGTs-catalyzed glucuronidation of 4-methylumbelliferone (4-MU) was used to investigate the inhibition of mangiferin and aglycone norathyriol towards various isoforms of UGTs in our study. The aim of this research was to investigate the inhibitory effects of mangiferin and aglycone norathyriol towards UGT isoforms, thus indicating potential UGT inhibition-related herb–drug interaction. Furthermore, molecular docking was used to elucidate the interactions between ligand and enzyme.

## 2. Results

### 2.1. Comparison of Inhibition Effect of MGF and NTR towards Various Important UGT Isoforms

**Figure 2:**
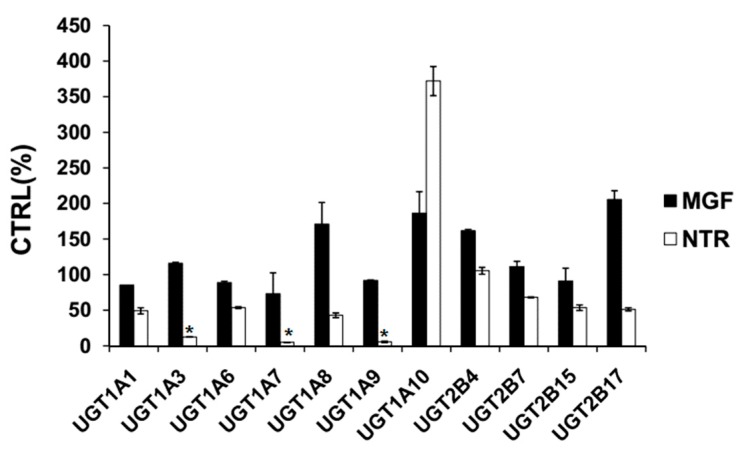
Screening the inhibition of UGT isoforms by 100 μM MGF and NTR. 4-methylumbelliferone (4-MU) was used as a probe substrate for recombinant human UGT1A1, UGT1A3, UGT1A6, UGT1A7, UGT1A8, UGT1A9, UGT1A10, UGT2B4, UGT2B7, UGT2B15, and UGT2B17, and data are shown using mean value plus SD. * *p* < 0.05.

Based on previous literature, 100 μM was chosen for MGF and NTR to screen the inhibition towards UGT isoforms [34,35]. As demonstrated in Figure 2, 100 μM MGF inhibited the activity of UGT1A1, 1A3, 1A6, 1A7, 1A8, 1A9, 1A10, 2B4, 2B7, 2B15, and 2B17 by 14.5%, −16.0%, 11.2%, 26.7%, −70.9%, 8.2%, −86.4%, −61.9%, −11.2%, 8.9%, and −105.6%, respectively. 100 μM NTR inhibited the activity of UGT1A1, 1A3, 1A6, 1A7, 1A8, 1A9, 1A10, 2B4, 2B7, 2B15, and 2B17 by 50.8%, 87.6%, 46.2%, 94.9%, 57.0%, 94.2%, −272.0%, −5.5%, 31.8%, 46.3%, and 48.6%, respectively. All these results showed that MGF and NTR both activated UGT1A10 and deglycosylation of MGF into NTR strongly increased the inhibitory effects towards others tested UGT isoforms except UGT 1A10.

### 2.2. Inhibition Type and Kinetics of NTR towards UGT1A3, UGT1A7 and UGT1A9

**Figure 3:**
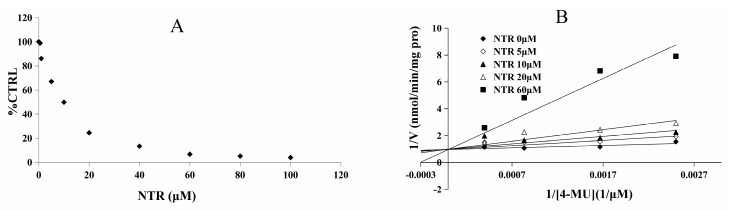
Determination of inhibition type and parameters (*Ki*) of NTR towards UGT1A3. (**A**) Dose-dependent inhibition of NTR towards UGT1A3; (**B**) Lineweaver-Burk plot of inhibition of NTR towards UGT1A.

**Figure 4:**
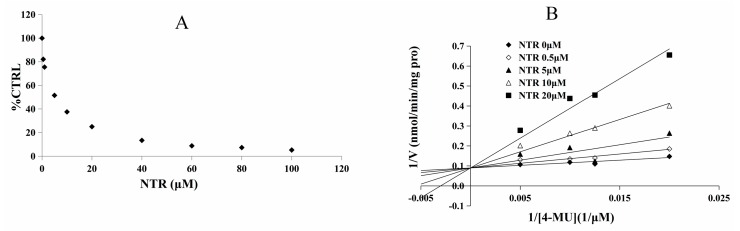
Determination of inhibition type and parameters (*Ki*) of NTR towards UGT1A7. (**A**) Dose-dependent inhibition of NTR towards UGT1A7; (**B**) Lineweaver-Burk plot of inhibition of NTR towards UGT1A7.

**Figure 5:**
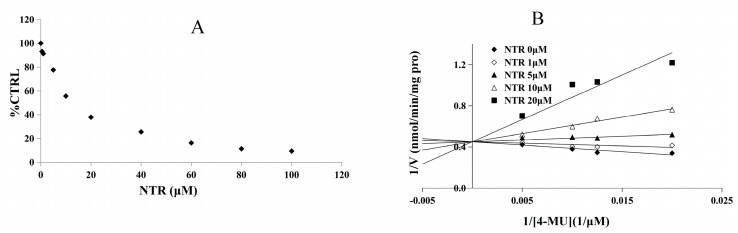
Determination of inhibition type and parameters (*Ki*) of NTR towards UGT1A9. (**A**) Dose-dependent inhibition of NTR towards UGT1A9; (**B**) Lineweaver-Burk plot of inhibition of NTR towards UGT1A9.

As the inhibitory capacity by NTR towards UGT1A3, UGT1A7 and UGT1A9 was above 80%, kinetic experiments were performed to further characterize the inhibition of these three UGT isoforms by NTR. The dose-dependent inhibition of NTR towards UGT1A3, UGT1A7 and UGT1A9 was shown in Figure 3A, Figure 4A and Figure 5A, with an IC_50_ value of 8.2, 4.4, and 12.3 μM, respectively. NTR competitively inhibited UGT1A3, UGT1A7 and UGT1A9, as demonstrated via Dixon plot and Lineweaver–Burk plot (Figure 3B, Figure 4B and Figure 5B). The inhibition constant (*Ki*) were calculated to be 1.6, 2.0, and 2.8 μM for the inhibition of NTR towards UGT1A3, UGT1A7 and UGT1A9, respectively.

### 2.3. In Silico Docking to Explain the Inhibition of NTR towards UGT1A3, UGT1A7 and UGT1A9

**Figure 6:**
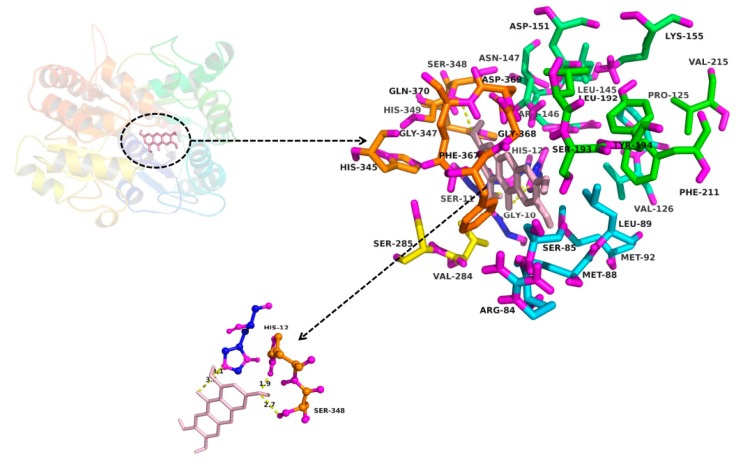
Homology modelling of the binding pocket for NTR in UGT1A3. The residues in the binding pocket were shown in stick. NTR was colored in gray. The formed hydrogen bonds between NTR and UGT1A3 were colored in yellow.

**Figure 7:**
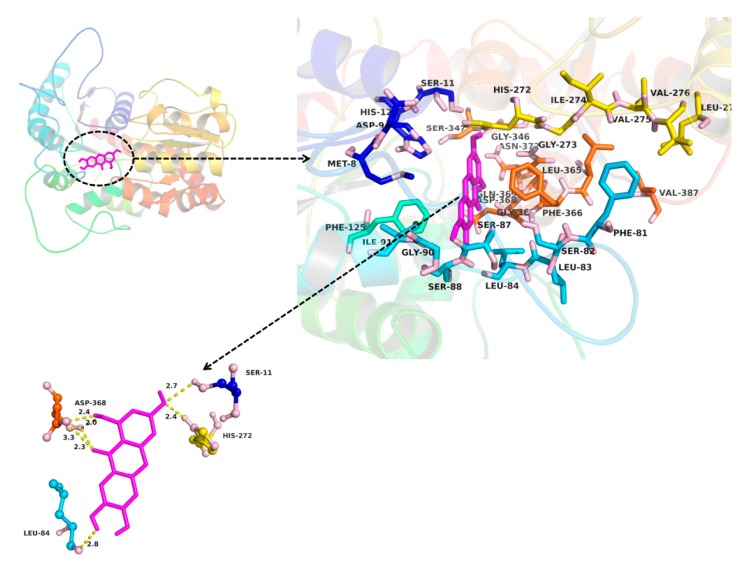
Homology modelling of the binding pocket for NTR in UGT1A7. The residues in the binding pocket were shown in stick. NTR was colored in magenta. The formed hydrogen bonds between NTR and UGT1A7 were colored in yellow.

**Figure 8:**
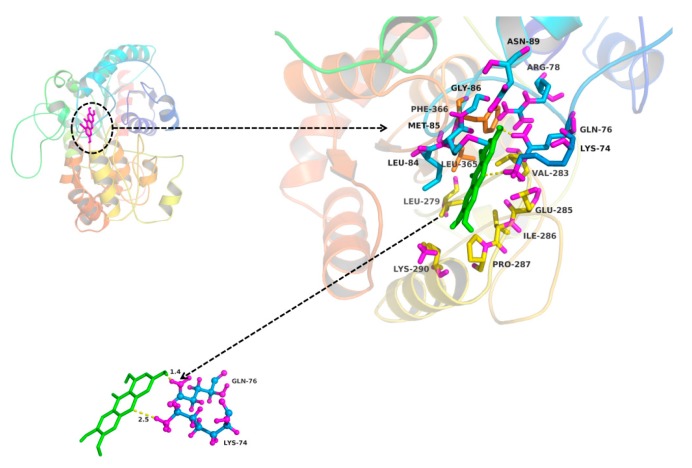
Homology modelling of the binding pocket for NTR in UGT1A9. The residues in the binding pocket were shown in stick. NTR was colored in green. The formed hydrogen bonds and hydrophobic interactions between NTR and UGT1A9 were colored in yellow.

The molecular docking method was used to explore the interaction between ligand (MGF and NTR) and protein (UGT1A3, UGT1A7 and UGT1A9). MGF could not dock into the activity cavity between N-terminal and C-terminal of UGT1A3, UGT1A7 and UGT1A9. Thus, only the binding pocket, the amino acid residues to form the activity cavity, the interactions and the binding free energy between NTR and UGT1A3, UGT1A7 and UGT1A9 were showed. The binding pocket of NTR for UGT1A3 was given in Figure 6. The amino acid residues to form the activity cavity contain Gly10, Ser11, His12, Arg84, Ser85, Leu89, Mer92, Pro125, Val126, Leu145, Arg146, Asn147, Asp151, Lys155, Leu192, Se r193, Tyr194, Pher211, Val284, Ser285, His345, Ser348, His349, Gly347, Asp369, Phe367, Gly368. Residues His12 and Ser348 made hydrogen bonds to NTR. The binding pocket of NTR for UGT1A7 was given in Figure 7. The amino acid residues to form the activity cavity contain Met8, Asp9, Ser11, His12, Phe81, Ser82, Leu83, Leu84, Ser87, Ser88, Gly90, Ile91, Phe125, His272, Gly273, Ile274, Val275, Vl276, Leu277, Gly346, Ser347, Leu365, Phe366, Gly367, Asp368, Gln369, Asn372, Val387. Residues Ser11, Leu84, His272 and Asp368 made hydrogen bonds to NTR. The binding pocket of NTR for UGT1A9 was given in Figure 8. The amino acid residues to form the activity cavity contain Lys74, Gln76, Artg78, Leu84, Mer85, Gly86, Asn89, Leu279, Val283, Glu285, Ile286, Pro287, Lys290, Leu365, Phe366. Residues Lys74 made hydrophobic interactions to NTR and Gln76 made hydrogen bonds to NTR. The binding free energies of NTR to UGT1A3, 1A7, 1A9 were −7.4, −7.9 and −4.0 kcal/mol, respectively.

## 3. Discussion

In our research, the inhibitory effects of MGF and NTR on UGT isoforms were studied and led to the conclusion that deglycosylation of MGF to NTR can strongly increase the inhibitory effects towards almost all tested UGT isoforms. Exogenous compounds have two ways of regulating enzymes, namely inhibition and activation. In our experiments, it was found that MGF and NTR activated UGT 1A10 and deglycosylation of MGF into NTR strongly increased the inhibitory effects towards others tested UGT isoforms except UGT1A10. The deglycosylation of saponin to aglycone always exhibited a different inhibition profile, such as astragaloside IV and cycloastragenol, glucoaurantio-obtusin and aurantio-obtusin, liquiritin and liquiritigenin, icariin and its intestinal metabolites (icariside I, icariside II and icaritin), arctiin and arctigenin, scutellarin and scutellarein, and in most situations, aglycone showed stronger inhibitory effects than saponin [33,34,35,36,37,38,39,40]. In our study, in silico docking method was used to explain why the inhibition potential of NTR was stronger than MGF on the activity of UGT1A3, UGT1A7 and UGT1A9. As shown in Figure 6, Figure 7 and Figure 8, several amino acid residues of these three UGT isoforms made hydrogen bonds or hydrophobic interactions to NTR. The binding free energy of NTR to UGT1A3, 1A7, 1A9 were −7.4, −7.9 and −4.0 kcal/mol, respectively. However, MGF could not dock into the activity cavity between N-terminal and C-terminal of UGT1A3, UGT1A7 and UGT1A9. The 1-OH and 3-OH groups in NTR played important role in making interactions to these three UGT isoforms, while MGF was the 2-C -glucosides of NTR, which generated stereo-hindrance effect compared with NTR, thus hindering the interactions between MGF and protein.

As exhibited in Figure 2, 100 μM NTR strongly inhibited the activity of UGT1A3, UGT1A7 and UGT1A9 by 87.6%, 94.9% and 94.2%, respectively. UGT1A3 is found in the liver, kidney, and prostate, and throughout the gastrointestinal tract [41]. UGT1A3 metabolizes endobiotic substances such as bile acid (CDCA, LCA, HDCA) and xenobiotic substances such as polyaromatic hydrocarbons amines, non-steroidal anti-inflammatory drugs (NSAIDs) and statins [42,43,44,45,46]. UGT1A7 represents one of several UGTs that were shown to be active on chemical carcinogens. UGT1A7 is an important extrahepatic UGT, and only presents in the esophagus, stomach, and lung [47]. UGT1A9 is one of the major enzymes responsible for the conjugation of carboxylic acids. UGT1A9 shows a wide spectrum of substrate specificity including bulky phenols, dietary constituents, steroids, and fatty acids, as well as prescribed drugs including anticancer agents, fibrates, NSAIDs, and antiarrhythmic agents [48].

In practice, the [I]/*Ki* ratio is used to predict the likelihood inhibitory drug-drug interactions. When assessing in vivo interaction potential, [I] represents the mean steady-state C_max_ value following the administration of the highest proposed clinical dose [49]. Extensive research into the pharmacokinetics of MGF in rat had been investigated. The maximum concentrations in the plasma were 8.13 ± 1.96 μg/mL (19.2 ± 4.64 μM) and 0.19 μg/mL (0.45 μM) after oral administration of MGF at a dose of 120 mg/kg and 70mg/kg in rat, respectively [12,50]. To the best of our knowledge, no one research on the pharmacokinetic study for mangiferin in human has been done. The concentration of mangiferin in plasma reached 38.64 ± 6.75 ng/mL (0.091 ± 0.016 μM) about 1 h after oral administration of 0.9 g mangiferin in human [51]. As demonstrated in Figure 6, Figure 7 and Figure 8, the inhibition constant (*Ki*) were calculated to be 1.6, 2.0, and 2.8 μM for the inhibition of NTR towards UGT1A3, UGT1A7 and UGT1A9, respectively. However, we cannot accurately predict in vivo inhibition magnitude only based on the above experimental results. Multiple factors should be taken into account when predict the likelihood inhibitory drug-drug interactions. First, the drug dose should be the highest proposed clinical dose. Secondly, the aglycone of MGF, rather than MGF, had the inhibition effect on UGTs, so the metabolism of MGF to NTR should also be considered. In fact, the NTR exposure after oral administration of MGF was extremely slow, and after administration of 200 mg/kg MGF, the concentration and exposure of NTR was quite low (C_max_ < 3 ng/mL), which was lower than that of the parent drug (C_max_: 202.58 ng/mL) by roughly two orders of magnitude (AUCNTR/AUCMGF < 3%) [52]. However, given of its slow generation and elimination rates, possible accumulation of NTR in the long term of MGF treatment and exert effects should be considered. Thirdly, the species difference has prevented their results from being directly applied to humans. Although complicated, study on the inhibition potential of MGF/NTR applied us more information for the MGF/NTR-drug interactions in clinical application.

In conclusion, the specific inhibition of MGF and its aglycone NTR was demonstrated in this manuscript. The deglycosylation of MGF into NTR strongly increased the inhibitory effects towards others tested UGT isoforms except UGT1A10. In silico prediction model was successfully employed to explain the different inhibition effects of MGF and NTR on UGT1A3, UGT1A7 and UGT1A9. The inhibition kinetic type and behavior were determined for the inhibition of NTR towards UGT1A3, UGT1A7 and UGT1A9, and using IVIVE to clarify the possibility of herb–drug interaction between MGF/NTR and drugs. Based on these results, clinical close monitoring the utilization of norathyriol is very important and necessary.

## 4. Materials and Methods

### 4.1. Chemicals

Mangiferin and norathyriol were purchased from BioBioPha Co. (Yunnan, China), and the purity of these two compounds was demonstrated to be above 95%. Sigma-Aldrich (St Louis, MO, USA) provided the following constituents in the incubation mixture: 4-methylumbelliferone (4-MU), 4-methylumbelliferone-β-d-glucuronide (4-MUG), Tris-HCl, 7-hydroxycoumarin, and uridine-diphosphoglucuronic acid (UDPGA) (trisodium salt). All other compounds were of high-performance liquid chromatography (HPLC) grade or of the highest grade commercially available. For enzyme sources, recombinant UGT isoforms expressed in baculovirus-infected insect cells were purchased from BD Gentest Corp. (Woburn, MA, USA), including UGT1A1, 1A3, 1A6, 1A7, 1A8, 1A9, 1A10, 2B4, 2B7, 2B15, and 2B17.

### 4.2. Investigation of the Inhibition Potential of MGF and NTR on UGT Isoforms

4-MU, a nonspecific probe substrate for all the UGT isoforms, was employed to investigate the inhibition of MEF and NIR towards the activity of UGT isoforms. The incubation and analytical methods have been previously described [53,54,55]. A typical 200 μL incubation mixture, contained various recombinant UGT isoforms (0.125 mg/mL for UGT1A1, 0.05 mg/mL for UGT1A3, UGT1A7, UGT1A9, UGT1A10 and UGT2B7, 0.025 mg/mL for UGT1A6 and UGT1A8, 0.25 mg/mL for UGT2B4, 0.2 mg/mL for UGT2B15, 0.5 mg/mL for UGT2B17), 5 mM UDPGA, 5 mM MgCl2, 50 mM Tris-HCl buffer (pH = 7.4), and various concentrations of 4-MU (110 μM for UGT1A1 and UGT1A6, 1200 μM for UGT1A3, 30 μM for UGT1A7, UGT1A9 and UGT1A10, 750 μM for UGT1A8, 1 000 μM for UGT2B4, 350 μM for UGT2B7, 250 μM for UGT2B15 and 2 000 μM for UGT2B17) in different concentration of MGF or NTR. MEF and NIR were dissolved in the DMSO to make a stock solution of 20 mM, and various concentrations of working solutions were prepared through dilution with DMSO. 4-MU was used as a non-selective substrate of UGTs. There was a 5-min pre-incubation step at 37 °C before the reaction was initiated by the addition of UDPGA. 4-MU and inhibitors were previously dissolved in DMSO, and the total concentration of DMSO was 1%. The reactions were continued at 37 °C for 120 min for UGT1A1, UGT1A3, UGT1A10, UGT2B4, UGT2B7, UGT2B15 and UGT2B17, 30 min for UGT1A6, UGT1A7, UGT1A8, and UGT1A9, respectively. Reactions were terminated by the addition of 100 μL acetonitrile with 7-hydroxycoumarin (100 μM) as internal standard. The incubation mixtures were then centrifuged at 12,000 r/min for 10 min. 2 μL supernatant was injected into the UPLC system for analysis. An ACQUITY UPLC System (Waters, Milford, MA, USA) equipped with UV detector was used to analyze the samples, and the separation of all the compounds were carried out using a ACQUITY UPLC^®^ BEH C18 (2.1 × 100 mm, 1.7 μm Waters) at a flow rate of 0.2 mL/min and UV detector at 316 nm. The mobile phase was consisted of ultrapure water containing 0.5% formic acid (A) and acetonitrile (B). The following gradient condition was used: 0–3.5 min, 10–65% B; 3.5–4.0 min, 65% B; 4.0–9.0 min, 10% B. To calculate the standard curve 0.1–100 μM of 4-MUG was used through drawing the peak area ration of 4-MUG/internal standard towards the concentration range of 4-MUG. The curve was linear over this concentration range, with an *r*^2^ value > 0.99.

### 4.3. In Silico Docking to Explain the Inhibition of NTR towards UGT1A3, UGT1A7 and UGT1A9

Comparative homology modeling with MODELLER9v14 program was carried out to elucidate the three-dimensional structure of UGT1A, UGT1A7 and UGT1A9, respectively [56,57]. The amino acid sequence of UGT1A3, UGT1A7 and UGT1A9 was downloaded from National Center for Biotechnology Information with the code NP_061966.1, NP_061950.2 and NP_066307.1, respectively. The crystal structure of human glucosyltransferase UGT78G1 (PDB code: 3hbf) has 33% identity in amino acid sequence with the C-terminal of UGT1A3, UGT1A7 and UGT1A9. Twenty comparative models of target sequence were built by MODELLER program, and the best model was selected based on the Modeller objective function and Discrete Optimization Protein Energy score. Auto-dock version 4.2 program was employed to perform the interaction between NTR and UGT1A3, UGT1A7 and UGT1A9, respectively [58,59]. Polar hydrogen atoms were added to UGT1A3, UGT1A7 and UGT1A9, and nonpolar hydrogenatoms were merged. AutoDock tool was used to add the Kollman charges to UGT1A3, UGT1A7 and UGT1A9. The grid box was generated with a dimension of 40 × 40 × 40. The grid spacing was set to 0.375 Å. The Lamarckian geneticalgorithm was utilized to deal with the protein-fixed ligand-flexible docking calculations. After docking calculation, the ligands were ranked according to the docked energy, and the best conformation with the lowest docked energy was selected to analyze the interactions between inhibitor and protein.

### 4.4. Inhibition Kinetic Analysis and In Vitro–In Vivo Extrapolation (IVIVE)

The inhibition type and Kinetics were determined for the inhibition of NTR towards UGT1A3, UGT1A7 and UGT1A9. The glucuronidation velocity of 4-MU was determined at multiple concentrations of 4-MU and NTR. Dixon and Lineweaver–Burk plots were employed to determine the inhibition type, and the second plot of the slopes from the Lineweaver–Burk plot versus the compound concentrations was utilized to calculate the *Ki* value. The determination of IC_50_ was calculated using Probit analysis in SPSS11.5 (SPSS, Chicago, IL, USA). The Student’s *t*-test was adopted at a significance level of *p* < 0.05 to determine statistically significant differences among experimental groups. In vitro-in vivo extrapolation (IVIVE) was performed using the following equation:

AUCi/AUC = 1 + [I]_in vivo_/*Ki*

The terms are defined as follows: AUCi/AUC is the predicted ratio of in vivo exposure of xenobiotics or endogenous substances with or without the co-exposure of CAG. [I]_in vivo_ is the in vivo exposure concentration of CAG, and the *Ki* value was in vitro inhibition constant. The evaluation standard was as follows: [I]/*Ki* < 0.1, low possibility; 0.1 < [I]/*Ki* < 1, medium possibility; [I]/*Ki* > 1, high possibility.
